# Sustained low-efficiency dialysis for renal replacement therapy in the ICU: a cost-benefit analysis of the years 2006 to 2010

**DOI:** 10.1186/cc10976

**Published:** 2012-03-20

**Authors:** T Neuenfeldt, HB Hopf

**Affiliations:** 1 Asklepios Klinik, Langen, Germany

## Introduction

Sustained low-efficiency dialysis (SLED) as primary renal replacement therapy in acute renal failure is still not widely used compared to continuous venovenous hemodiafiltration (CVVHDF), despite possible economical advantages. Based on one key paper [1] we use SLED as primary renal replacement therapy. However, since medical and economical data with SLED are scarce, we evaluated costs and outcome in a 5-year retrospective study on our ICU.

## Methods

During 2006 to 2010 we performed a search on our KIS selecting all patients with the diagnoses N17 and N18 who were treated with SLED or CVVHDF on our ICU. We excluded all patients with a stay <2 days or with an extrarenal indication for dialysis or with pre-existing chronic dialysis. The following variables were extracted from the chart: number of SLED, stay in ICU and hospital, mortality in ICU and hospital, SAPS II, TISS 28, blood urea and creatinine, C-reactive protein, mechanical ventilation, and diagnoses. We evaluated the long-term outcome by sending all discharged patients a questionnaire.

## Results

**Figure 1:**
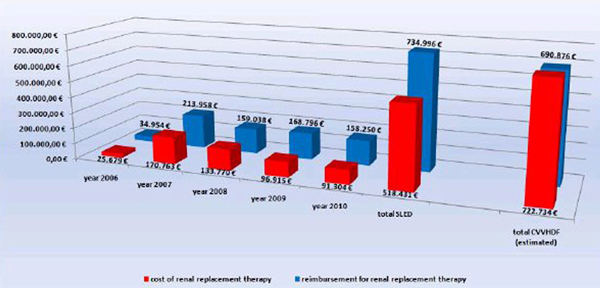
**Cost-benefi t ratio for SLED compared to CVVHDF**.

During the period from 2006 to 2010, 3,247 SLED treatments in 421 patients (mean SAPS II was 52 patients) were performed. ICU mortality was 36% and hospital mortality was 46%. A persistent need for dialysis (end-stage kidney disease) was registered in 9%. Total costs for SLED were €518.431 and total reimbursements amount to €734.996 (Figure 1). Assuming for cost comparisons also 3,247 CVVHDF-days, we estimated costs of €722.734 with reimbursements of €690.876 for CVVHDF.

## Conclusion

Thus, since short-term and long-term outcome of our patients was comparable to published outcome data with CVVHDF, SLED is at least comparable to CVVHDF even in a busy ICU environment. Moreover, in view of costs, SLED is the preferable dialysis form for renal replacement therapy also in the ICU.
